# Endometrial Volume Measured by VOCAL Compared to Office Hysteroscopy for Diagnosis of Endometrial Polyps in Premenopausal Women with Abnormal Uterine Bleeding

**DOI:** 10.1155/2016/3561324

**Published:** 2016-11-24

**Authors:** Mohamed Laban, Sherif H. Hussain, Alaa S. Hassanin, Waleed M. Khalaf, Mohamed K. Etman, Mohammed S. E. Elsafty, Ahmed M. Bahaa Eldin, Ahmad S. Hasanien, Noha A. Sakna, Mohammed Taema, Mohammed H. Mostafa, Marwa M. Eisa

**Affiliations:** ^1^Department of Obstetrics and Gynecology, Faculty of Medicine, Ain Shams University, Cairo, Egypt; ^2^Fetal Special Care Unit, Ain Shams Maternity Hospital, Faculty of Medicine, Ain Shams University, Cairo, Egypt; ^3^Murwillumbah Hospital, Murwillumbah, NSW, Australia

## Abstract

The aim is to compare hysteroscopy, two-dimensional transvaginal ultrasound (2D TVUS), and three-dimensional (3D) Virtual Organ Computer-aided AnaLysis™ (VOCAL) to detect endometrial polyps (EPs) in premenopausal women with abnormal uterine bleeding (AUB). This prospective study was done at Ain Shams Maternity Hospital, Egypt, from March 5, 2015, to December 30, 2015, enrolling 118 premenopausal women with AUB. 2D TVUS, 3D VOCAL, and hysteroscopy were done. 109 patients reached final analysis. 36 women (33%) were diagnosed with EP by 2D TVUS. 50 (45.9%) had EP by hysteroscopy. Endometrial thickness was 10.1 mm by 2D TVUS and endometrial volume was 4.92 mL by VOCAL in women with EP by hysteroscopy compared to 9.9 mm and 3.50 mL in women with no EP, respectively (*P* = 0.223; *P* = 0.06). 2D TVUS has sensitivity, specificity, and positive and negative predictive values of 54%, 84.7%, 75%, and 68.5%, respectively. Endometrial thickness of >7.5 mm has sensitivity, specificity, positive and negative predictive values, and overall accuracy of 82%, 37.3%, 52.6%, 71%, and 57.8%, respectively. Endometrial volume of >1.2 mL has sensitivity, specificity, positive and negative predictive values, and overall accuracy of 90%, 42.4%, 57%, 83.3%, and 64.2%, respectively. 3D VOCAL may be used as a noninvasive method for the diagnosis of EP in premenopausal women with AUB.

## 1. Introduction

Endometrial polyps (EPs) are benign growths of the endometrium appearing as a localized swelling protruding into the uterine cavity. Histologically, an endometrial polyp consists of endometrial glands surrounded by stroma and covered by epithelium [1]. It is a common gynecological condition in premenopausal women with 16% prevalence in asymptomatic women of the childbearing age [2, 3]. The condition is even more prevalent in symptomatic women, up to 34%. Symptoms are usually in the form of abnormal uterine bleeding (AUB) or infertility [4].

Various tools for diagnosis are two-dimensional transvaginal ultrasound (2D TVUS), sonohysterography, hysteroscopy, and curettage followed by histopathology. Hysteroscopy is the gold standard for the confirmation of the diagnosis enabling the gynecologist not only to detect the EP, its site, and number but also to remove it in the same setting [5, 6].

Endometrial polyps may appear as a thick regular endometrium by 2D TVUS or as focal masses within the endometrial canal. Conventional 2D TVUS is useful in providing information about the uterus through axial and sagittal cuts, yet quite limited in the coronal plane. On the other hand, 3D TVUS overcomes this limitation by reconstructing the coronal plane of the uterus, providing more accurate diagnosis of uterine anomalies and higher definition of endometrial delineation, hence higher accuracy of localization of endometrial polyps, fibroids, and intrauterine devices [7, 8]. However, one limitation of this advanced technology is that there is great discrepancy between sonographers in their ability to differentiate between a normal and abnormal uterine cavity. To overcome such limitation, increase diagnostic accuracy of measurement of 3D volumes, and decrease interobserver discrepancy, it is mandatory to utilize a standard technique or program for analyzing and calculating 3D volumes [9].

Measurement of endometrial volume by Virtual Organ Computer-aided AnaLysis (VOCAL) has been widely studied [10–16]; however, no study has proposed a cut-off value for endometrial volume of symptomatic premenopausal women with AUB caused by endometrial polyps.

The current study is the first to date addressing the use of VOCAL imaging program to measure endometrial volume for improving the accuracy of diagnosing EPs in symptomatic premenopausal women with AUB over the conventional 2D TVUS in comparison to hysteroscopy.

## 2. Materials and Methods

This prospective study was carried out at Ain Shams Maternity Hospital, Cairo, Egypt, from 5 March 2015 to 30 December 2015. 118 premenopausal women complaining of abnormal uterine bleeding in the form of menorrhagia, metrorrhagia, or both were recruited. Menopausal women, women with general causes of bleeding like bleeding disorders and liver diseases, and women on drugs like hormonal contraception and anticoagulants were excluded; so were patients with fibroids and fibroid polyps. This study conforms to the Declaration of Helsinki and was registered on clinicaltrials.gov with registration number NCT02300805. Institutional review board approval was obtained on 10 November 2014 and all women signed written informed consent at recruitment.

Two-dimensional transvaginal ultrasound, Virtual Organ Computer-aided AnaLysis (VOCAL) imaging program, and diagnostic hysteroscopy were done for all women for detection of endometrial polyps.

Two-dimensional transvaginal ultrasound was done using Voluson E6 (General Electric Healthcare, USA) S-VDW 5–8 MHz transvaginal probe to measure endometrial thickness after obtaining longitudinal plane of the uterus and to detect the presence of endometrial polyps.

VOCAL imaging program was used for all patients to measure endometrial volume. Voluson E6 (General Electric Healthcare, USA) three-dimensional (3D) system with RIC 5-9 D transvaginal probe was used. A longitudinal view of the uterus was obtained and “Volume Analysis” on the touch panel was selected, and then VOCAL was selected. “Reference Image: A” was activated. The endometrium was adjusted to fit within green arrows and manually traced by using trackball/pointer. For a valid trace, the trace pointer had to cross the rotation axis line twice. The pointer was moved to the edge of the endometrium and then moved to a new area to be traced. Six traces were drawn by rotating at 30°. Sequence was repeated for all traces. On the final trace, “Done” and then “Accept Region of Interest (ROI)” were selected to obtain endometrial volume in milliliters. All ultrasound scans were done by a single highly experienced operator (fifth author).

Outpatient office hysteroscopy was done 1 to 3 days after the ultrasonography. It was performed using a 2.9 mm telescope with a 30° fore oblique lens (Karl Storz GmbH, Tuttlingen, Germany) inserted in a continuous flow 4 mm sheath (Sopro-Comeg GmbH, Tuttlingen, Germany). Isotonic saline was used as distension medium in all cases. Introduction was by the nontouch vaginoscopic technique without anesthesia in most cases, inspecting the vagina, cervix, and uterine cavity. Cases with difficult vaginoscopic entry were done conventionally using Collin's speculum and tenaculum. If polyps were found, they were simultaneously excised using 5 Fr. hysteroscopic scissors (Sopro-Comeg GmbH, Tuttlingen, Germany) or a bipolar electrode needle (Ackermann Instrumente GmbH, Germany).

## 3. Sample Size Calculation

The required sample size was estimated using the Power Analysis and Sample Size software version 11.0.10 (PASS, NCSS statistical software, LLC, Kaysville, Utah, USA). According to a previous study [2], the prevalence of endometrial polyps in premenopausal women with abnormal uterine bleeding was 33%. Using a two-sided binomial test with a confidence level of 95% (type I error 0.05) and an assumed prevalence of endometrial polyps of 33%, a sample size of 109 patients was calculated to achieve a power of 82% (type II error 0.18) to detect a statistically significant difference.

Data were analyzed using IBM SPSS Statistics version 22 (IBM Corp., Armonk, NY, USA). The Shapiro-Wilk test was used to examine the normality of numerical data distribution. Normally distributed numerical data were presented as mean ± SD and intergroup differences were compared using the unpaired *t*-test. Skewed numerical variables were presented as median (interquartile range) and between-group differences were compared using the Mann–Whitney test. Categorical data were presented as number (%) and between-group differences were compared using Fisher's exact test. Ordinal data were compared using the chi-squared test for trend. Receiver-operating characteristic (ROC) curve analysis was used to examine the value of endometrial thickness as estimated with 2D TVUS or endometrial volume as estimated with VOCAL in differentiating between patients with or without histopathologically diagnosed endometrial polyps. The DeLong method was used to compare the area under the ROC curve versus that of random prediction. *P* value <0.05 was considered statistically significant.

## 4. Results

118 patients were recruited but data of 109 patients reached final statistical analysis. Five patients were excluded because of diagnosis of multiple uterine fibroids by initial 2D TVUS and another four women were excluded because of failure of visualization of the uterine cavity during hysteroscopy because of excessive uterine bleeding.

**Figure 1:**
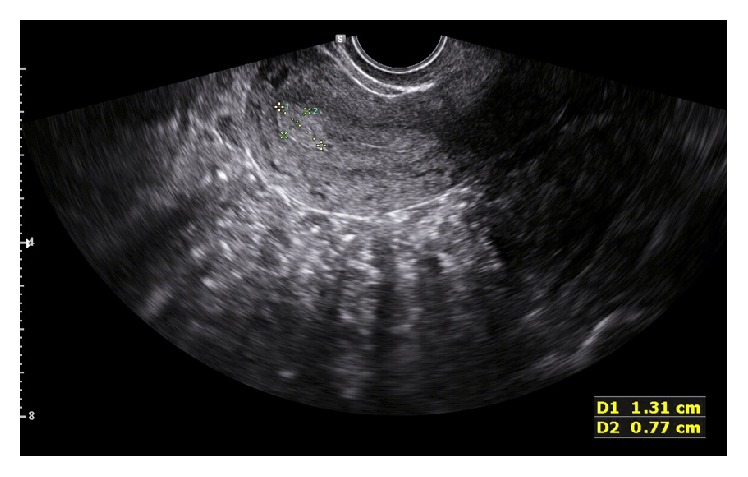
Endometrial polyp identified by 2D TVUS in one of the participants.

The average age of participants was 35 years (interquartile range between 29.8 and 44 years). Their parity ranged from nullipara to para 7. 36 women (33%) were initially diagnosed with endometrial polyps by 2D TVUS (Figure 1). 50 (45.9%) women were confirmed to have endometrial polyps by hysteroscopy.

**Figure 2:**
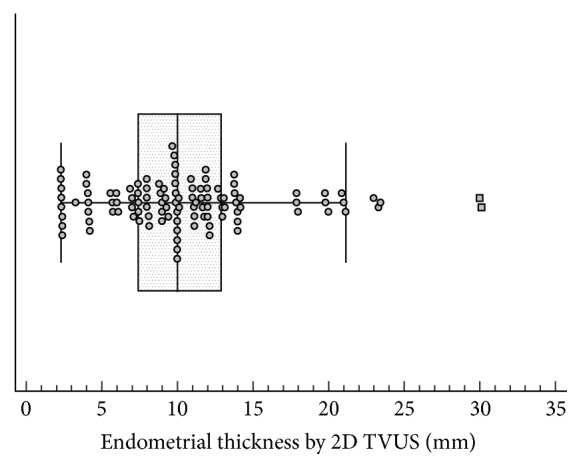
Box plot showing the distribution of values of endometrial thickness of the participants measured by 2D TVUS. Markers represent individual observations. Box represents the interquartile range. Line inside the box represents the median. Whiskers represent the minimum and maximum values excluding outliers and extreme observations.

**Figure 3:**
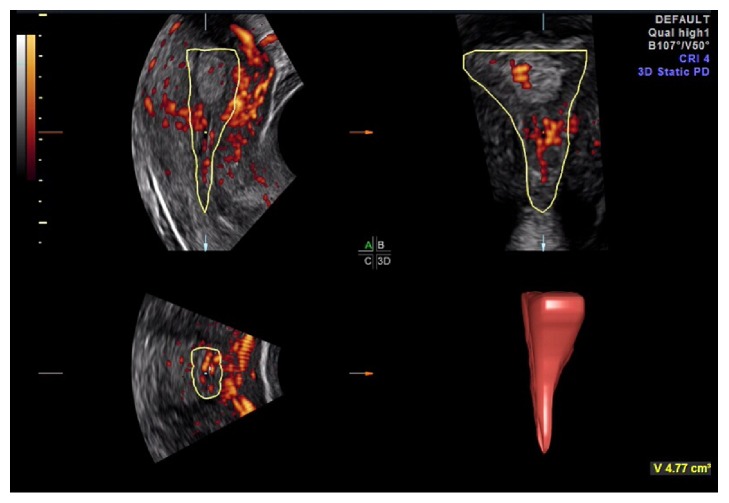
Endometrial volume measured by VOCAL for one of the participants.

**Figure 4:**
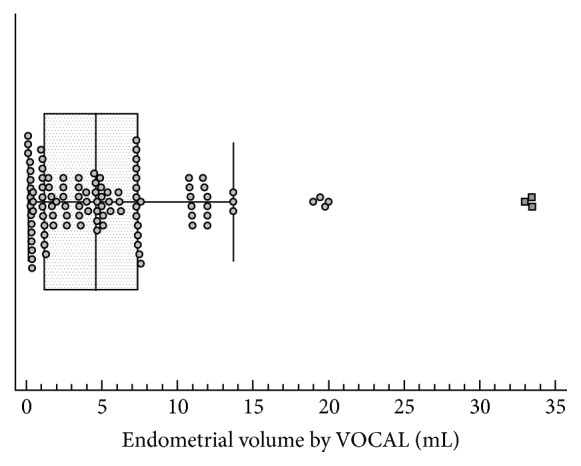
Box plot showing the distribution of the values of endometrial volume of the participants measured by VOCAL. Markers represent individual observations. Box represents the interquartile range. Line inside the box represents the median. Whiskers represent the minimum and maximum values excluding outliers and extreme observations.

Average endometrial thickness by 2D TVUS was 10 mm (range between 7.4 mm and 12.9 mm) (Figure 2). Average endometrial volume obtained by VOCAL was 4.59 mL (range between 1.18 mL and 7.39 mL) (Figures 3 and 4 and Table 1).

Out of the 50 women confirmed to have EP by hysteroscopy, 40 women (80%) had single polyp; 7 women (14%) had 2 or 3 polyps; and 3 women (6%) had more than 3 polyps. Mean size of the polyps was 11.8 mm (range from 7.3 mm to 19.6 mm). Histopathology results showed that 9 women (18%) had functional polyps; 18 women (36%) had hyperplastic polyps; 14 women (28%) had mucous polyps; and 9 women (18%) had proliferative polyps.

**Figure 5:**
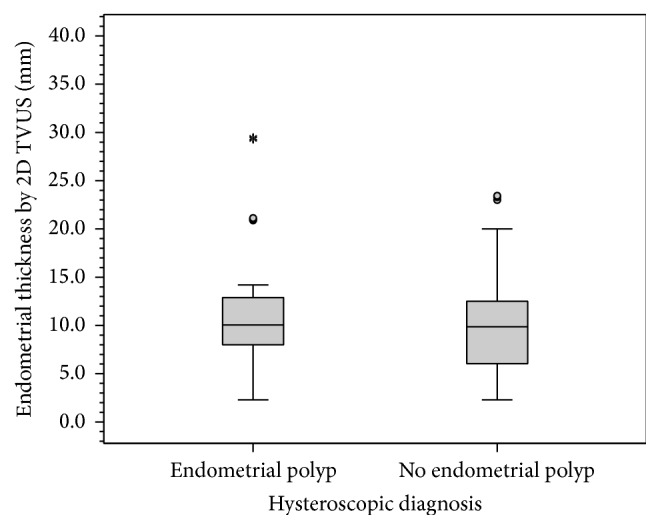
Box plot showing values of endometrial thickness measured by 2D TVUS in patients with or without endometrial polyps. Box represents the interquartile range. Line inside the box represents the median. Whiskers represent the minimum and maximum values excluding outliers (rounded markers) and extreme observations (asterisks).

**Figure 6:**
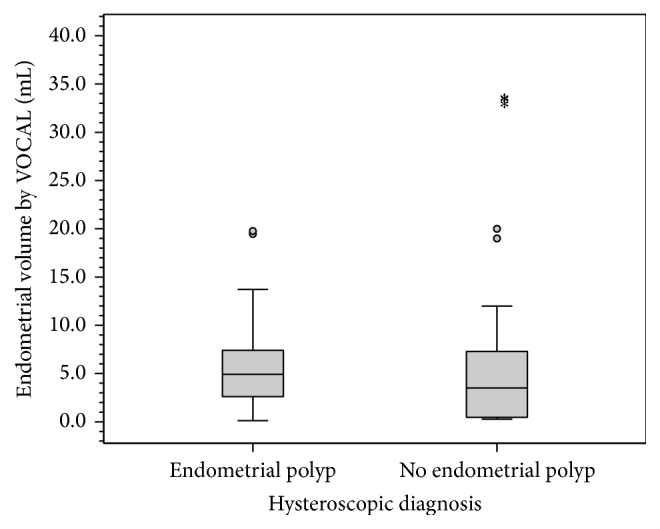
Box plot showing values of endometrial volume measured by VOCAL in patients with or without endometrial polyps. Box represents the interquartile range. Line inside the box represents the median. Whiskers represent the minimum and maximum values excluding outliers (rounded markers) and extreme observations (asterisks).

Comparing women with abnormal uterine bleeding diagnosed with or without endometrial polyps using hysteroscopy, there was no statistical difference regarding age and parity (*P* = 0.435 and *P* = 0.282, resp.). Average endometrial thickness by 2D TVUS was 10.1 mm in women diagnosed with endometrial polyp using hysteroscopy compared to 9.9 mm in women with no endometrial polyp (*P* = 0.223) (Figure 5 and Table 2). Endometrial volume by VOCAL was 4.92 mL in women diagnosed with endometrial polyps compared to 3.50 mL in women without. This statistical difference was insignificant (*P* = 0.062) (Figure 6 and Table 2).

Diagnostic accuracy of 2D TVUS to detect endometrial polyps compared to hysteroscopy which is the gold standard diagnostic tool is shown in Table 3. Sensitivity and specificity are 54% and 84.7%, respectively. Positive and negative predictive values are 75% and 68.5%, respectively.

**Figure 7:**
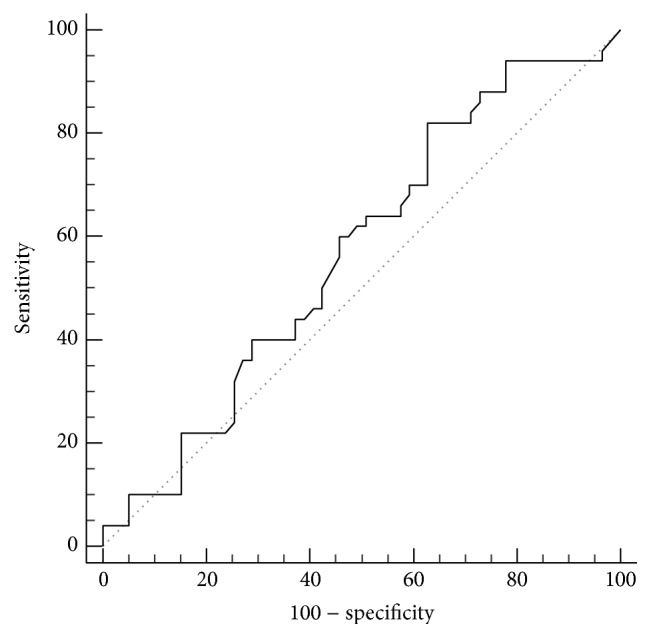
Receiver-operating characteristic (ROC) curve for differentiating between patients with or without endometrial polyps using endometrial thickness measured by 2D TVUS.

Table 4 and Figure 7 show the results of receiver-operating characteristic (ROC) curve analysis for the value of endometrial thickness by 2D TVUS in differentiating between patients with or without hysteroscopically diagnosed endometrial polyps. Endometrial thickness by 2D TVUS has an area under curve (AUC) of 0.568, which is not statistically significant compared to random prediction (*P* = 0.219). An endometrial thickness of >7.5 mm has a sensitivity of 82.0%, a specificity of 37.3%, and an overall accuracy of 57.8%. Positive and negative predictive values are 52.6% and 71%, respectively.

**Figure 8:**
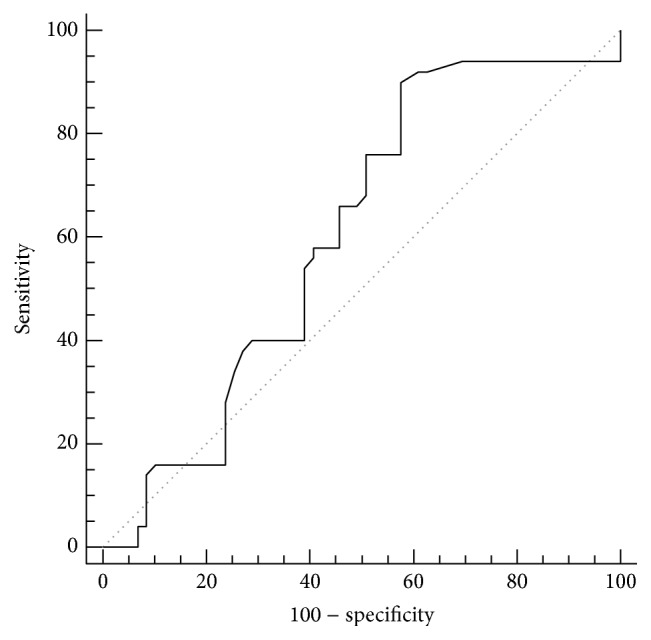
Receiver-operating characteristic (ROC) curve for differentiating between patients with or without endometrial polyps using the endometrial volume measured by VOCAL.

Table 5 and Figure 8 show the results of ROC curve analysis for the value of endometrial volume by VOCAL in differentiating between patients with or without hysteroscopically diagnosed endometrial polyps. Endometrial volume by VOCAL had AUC of 0.604, which was statistically insignificant compared to random prediction (*P* = 0.060). Endometrial volume of >1.2 mL has a sensitivity of 90%, a specificity of 42.4%, and an overall accuracy of 64.2%. Positive and negative predictive values are 57% and 83.3%, respectively.

## 5. Discussion

In the current study, an endometrial volume of more than 1.2 mL to predict endometrial polyps proved to be better than diagnosis by 2D TVUS and endometrial thickness of more than 7.5 mm. Sensitivity and specificity of 2D TVUS to diagnose endometrial polyps were 54% and 85%, respectively, while positive and negative predictive values were 75% and 69%, respectively. In comparison, cut-off value for endometrial thickness of more than 7.5 mm measured by 2D TVUS showed AUC of 0.568 which was statistically insignificant (*P* = 0.219). This endometrial thickness had a sensitivity and specificity of 82% and 37% with overall accuracy of 58%. PPV and NPV were 53% and 71%, respectively. On the other hand, cut-off value for endometrial volume measured by VOCAL of more than 1.2 mL was still statistically insignificant (*P* = 0.06) for AUC of 0.604. Sensitivity and specificity were 90% and 42% with overall accuracy of 64%, while PPV and NPV were 57% and 83%, respectively, which are superior to 2D TVUS.

De Godoy Borges et al. [17] studied the diagnostic accuracy of TVUS compared to hysteroscopy in diagnosis of EP in postmenopausal patients. TVUS showed sensitivity and specificity of 89% and 25%, respectively, with overall accuracy of 75%, while PPV and NPV were 82% and 38%. They concluded that hysteroscopy was more accurate than TVUS in diagnosing EP. The current study has also shown that 2D TVUS has poor diagnostic accuracy for detecting EP in premenopausal women. On the other hand, Dreisler et al. [18] studied the diagnostic accuracy of measuring endometrial thickness by TVUS for identifying EP in asymptomatic premenopausal women. A cut-off value of 5 mm had a positive predictive value of 10% and negative predictive value of 99%, concluding that endometrial thickness has poor diagnostic accuracy when diagnosing EP. Van Den Bosch et al. [9] also studied the diagnostic accuracy of measuring four different endometrial volumes using 3D TVUS to predict the presence of an intrauterine cavitary lesion in 111 women complaining of abnormal uterine bleeding regardless of their menopausal status. Four endometrial volumes (unenhanced TVUS and gel infusion TVUS with and without power Doppler) were obtained for every patient and six sonographers analyzed the offline images. The aim of their study was to detect the agreement of ultrasound diagnosis between each of the six sonographers and to measure the agreement between each sonographer's ultrasound diagnosis and the histological diagnosis. There was an agreement of 67% to 83% between ultrasound diagnosis and histological diagnosis for all six sonographers. They concluded that accuracy of diagnosis of an intracavitary uterine pathology by analyzing 3D volumes of the uterus varied significantly between sonographers due to lack of clinical data of the patients and decreased experience of the sonographer, in addition to suboptimal offline ultrasound images. Yet, in the current study, a single experienced sonographer has performed all scans; therefore, accuracy of 3D TVUS in diagnosing intrauterine polyps lacked interobserver variability. In addition, volume angle used in the previous trial was 120° compared to 30° in the current study and endometrial volume was measured using VOCAL.

Fang et al. [19] enrolled 426 asymptomatic infertile women to diagnose endometrial polyps in infertility using 2D TVUS diagnosis and endometrial thickness in addition to measurement of endometrial volume by VOCAL measured in postmenstrual days 3 to 7. They found that endometrial thickness and endometrial volume were higher in patients who were hysteroscopically diagnosed with EP. Sensitivity, specificity, and positive and negative predictive values of endometrial thickness at a cut-off value of 9.5 mm and endometrial volume at a cut-off value of 4.1 cm^3^ were 63%, 70%, 27%, and 92% and 39%, 88%, 36%, and 90%, respectively; when combining both parameters, ultrasound diagnostic accuracy improved to 66%, 89%, 50%, and 94%. In the current study, cut-off values for endometrial thickness and volume were different from those of Fang et al.; the latter study has recruited women with infertility rather than abnormal uterine bleeding with an 11% incidence of EP in Fang et al.'s study sample compared to 46% incidence of EP in the sample of the current study.

One strength of the current study is that it is the first to date addressing the diagnostic accuracy of VOCAL in prediction of endometrial polyps specifically in premenopausal women with AUB. Furthermore, it is the first study to provide a cut-off value for endometrial volume in premenopausal women with AUB. In addition, a single experienced sonographer performed all 2D and 3D TVUS which was done prior to hysteroscopy to avoid bias in diagnosis by ultrasound.

## 6. Conclusion

3D VOCAL has better diagnostic accuracy for EP in premenopausal women with AUB compared to 2D TVUS. 3D VOCAL may be used as a noninvasive preliminary method for diagnosis of premenopausal women complaining of AUB with EP who are in need for further uterine cavity assessment by hysteroscopy.

## Figures and Tables

**Table 1:** Data of all participants.

Variable	Value
Age (years)	35 (29.8–44.0)
Parity	
Para 0 to para 3	90 (82.6%)
Para 4 to para 7	19 (17.4%)
Endometrial polyps by 2D TVUS	36 (33%)
Endometrial thickness by 2D TVUS (mm)	10.0 (7.4–12.9); range: 2.3–30.1
Endometrial volume by VOCAL (mL)	4.59 (1.18–7.39); range: 0.12–33.5
Endometrial polyp by hysteroscopy	50 (45.9%)

Data are median (interquartile range) or number (%).

**Table 2:** Data of women with and without endometrial polyps diagnosed by hysteroscopy.

Variable	Endometrial polyps by hysteroscopy (*n* = 50)	No endometrial polyps by hysteroscopy (*n* = 59)	*t*/*χ* ^2^/*U*	DF/*Z*	*P* value
Age	36.6 ± 8.0	35.3 ± 9.1	−0.783	107	0.435
Parity			1.158	1	0.282
Para 0 to para 3	41 (82.0%)	49 (83.0%)			
Para 4 to para 7	9 (18.0%)	10 (17.0%)			
Endometrial thickness by 2D TVUS (mm)	10.1 (8.0–12.9)	9.9 (6.0–12.8)	1274.50	1.219	0.223
Endometrial volume by VOCAL (mL)	4.92 (2.60–7.40)	3.50 (0.46–7.30)	1168.50	1.864	0.062

Data are presented as mean ± SD, number (%), or median (interquartile range).

**(a):** 

2D TVUS	Hysteroscopy		
	*Polyp*	*No polyp*	*Total*
*Polyp*	27	9	36
*No polyp*	23	50	73
*Total*	50	59	109

**(b):** 

Statistical parameter	Value	95% confidence interval
Correct classification	70.6%	62.1%–79.2%
Misclassification	29.4%	20.8%–37.9%
Sensitivity	54.0%	40.4%–67.0%
Specificity	84.7%	73.2%–91.9%
False positive rate	15.3%	6.4%–24.1%
False negative rate	46.0%	32.7%–59.3%
Prevalence	45.9%	36.5%–55.2%
Positive predictive value (PPV)	75.0%	60.9%–89.1%
Negative predictive value (NPV)	68.5%	57.8%–79.1%
Positive likelihood ratio (LR+)	3.54	1.84–6.81
Negative likelihood ratio (LR−)	0.54	0.39–0.75

Data in contingency table are numbers of patients.

**(a):** 

Variable	Value
Sample size	109
Endometrial polyp by hysteroscopy	50 (45.87%)
No endometrial polyp by hysteroscopy	59 (54.13%)
Disease prevalence	45.9%

**(b):** 

ROC index	Value	95% confidence interval
Area under the ROC curve (AUC)	0.568	0.470 to 0.663
*z* statistic	1.229	
*P* value (AUC_0_ = 0.5)^a^	0.219	
Cut-off criterion	>7.5 mm	
Youden index (*J*)	0.1929	
Accuracy	57.8%	
Sensitivity	82.0%	68.6%–91.4%
Specificity	37.3%	25.0%–50.9%
Positive likelihood ratio (+LR)	1.3	1.0–1.7
Negative likelihood ratio (−LR)	0.5	0.2–1.0
Positive predictive value (+PV)	52.6%	40.9%–64.0%
Negative predictive value (−PV)	71.0%	52.0%–85.9%

^a^DeLong method.

**Table 5:** Receiver-operating characteristic (ROC) curve analysis for the value of endometrial volume as estimated with VOCAL in discrimination between patients with or without hysteroscopically diagnosed endometrial polyps.

ROC index	Value	95% confidence interval
Area under the ROC curve (AUC)	0.604	0.506–0.696
*z* statistic	1.881	
*P* value (AUC_0_ = 0.5)^a^	0.060	
Cut-off criterion	>1.2 mL	
Youden index (*J*)	0.324	
Accuracy	64.2%	
Sensitivity	90.0%	78.2%–96.7%
Specificity	42.4%	29.6%–55.9%
Positive likelihood ratio (+LR)	1.56	1.2–2.0
Negative likelihood ratio (−LR)	0.24	0.10–0.6
Positive predictive value (+PV)	57.0%	45.3%–68.1%
Negative predictive value (−PV)	83.3%	65.3%–94.4%

^a^DeLong method.
